# Metformin inhibits chronic kidney disease‐induced DNA damage and senescence of mesenchymal stem cells

**DOI:** 10.1111/acel.13317

**Published:** 2021-02-01

**Authors:** Hyoungnae Kim, Mi Ra Yu, Haekyung Lee, Soon Hyo Kwon, Jin Seok Jeon, Dong Cheol Han, Hyunjin Noh

**Affiliations:** ^1^ Department of Internal Medicine Soon Chun Hyang University Seoul Korea; ^2^ Hyonam Kidney Laboratory Soon Chun Hyang University Seoul Korea

**Keywords:** chronic kidney disease, DNA damage, mesenchymal stem cells, metformin, senescence

## Abstract

Mesenchymal stem cells (MSCs) are promising source of cell‐based regenerative therapy. In consideration of the risk of allosensitization, autologous MSC‐based therapy is preferred over allogenic transplantation in patients with chronic kidney disease (CKD). However, it remains uncertain whether adequate cell functionality is maintained under uremic conditions. As chronic inflammation and oxidative stress in CKD may lead to the accumulation of senescent cells, we investigated cellular senescence of CKD MSCs and determined the effects of metformin on CKD‐associated cellular senescence in bone marrow MSCs from sham‐operated and subtotal nephrectomized mice and further explored in adipose tissue‐derived MSCs from healthy kidney donors and patients with CKD. CKD MSCs showed reduced proliferation, accelerated senescence, and increased DNA damage as compared to control MSCs. These changes were significantly attenuated following metformin treatment. Lipopolysaccharide and transforming growth factor β1‐treated HK2 cells showed lower tubular expression of proinflammatory and fibrogenesis markers upon co‐culture with metformin‐treated CKD MSCs than with untreated CKD MSCs, suggestive of enhanced paracrine action of CKD MSCs mediated by metformin. In unilateral ureteral obstruction kidneys, metformin‐treated CKD MSCs more effectively attenuated inflammation and fibrosis as compared to untreated CKD MSCs. Thus, metformin preconditioning may exhibit a therapeutic benefit by targeting accelerated senescence of CKD MSCs.

## Graphical abstract

Increased senescence of CKD MSCs may contribute to poor regenerative potential. Metformin decreased CKD‐induced prelamin A accumulation and DNA damage signaling, which may lead to cellular senescence. We suggest metformin preconditioning as an effective strategy to overcome the senescence‐associated barrier to autologous patient‐derived MSC therapy in CKD.

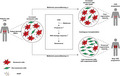

## 1 INTRODUCTION

Chronic kidney disease (CKD) is a global health burden that affects 10%–15% of the general population (Bikbov et al., 2020; Glassock et al., 2017). Studies are investigating cell‐based regenerative therapy as a treatment modality to stop the progression of CKD to end‐stage renal disease (Chung, 2019; Perico et al., 2018). Evidence suggests the promising role of mesenchymal stem cells (MSCs) for cell‐based therapies. MSCs can be obtained from various tissues, including the bone marrow, adipose tissue, skin, and skeletal muscle, and easily expanded in vitro. MSCs are multipotent and can differentiate into adipocytes, osteocytes, and chondrocytes (Dominici et al., 2006). Further, MSCs may accelerate tissue repair via a paracrine mechanism leading to proangiogenic, anti‐inflammatory, and antifibrotic effects than directly differentiating into tissue‐specific cells (Gnecchi et al., 2005). Preclinical and clinical trials are in progress to evaluate the beneficial effects of MSC therapy in various kidney disease models (Fazekas & Griffin, 2020; Perico et al., 2018). However, it is imperative to consider that autologous cell transplantation is preferred over allogenic transplantation in patients with CKD who may need kidney transplantation in the future, as the latter may increase the risk of allosensitization. Therefore, adequate functionality of endogenous MSCs is a critical factor for the success of cell therapies in patients with CKD.

We and others have shown that MSCs obtained from patients or rodents with CKD exhibit decreased viability and functional abnormalities (Klinkhammer et al., 2014; Noh et al., 2012, 2014). Moreover, under uremic milieu, the angiogenic potential of transplanted MSCs was significantly reduced at ischemic hind limb (Han et al., 2019; Noh et al., 2014). In the present study, we investigated the effects of metformin on the functional incompetence of CKD MSCs, particularly by focusing on its renoprotective potential. Metformin is a first‐line drug for the treatment of type 2 diabetes (Davies et al., 2018). Aside from its anti‐diabetic effect, metformin is known to exert pleiotropic actions, including beneficial effects on the kidney and cardiovascular system and by possibly lowering cancer risk (Foretz et al., 2014). The renal protective effects of metformin have been demonstrated in multiple disease models such as acute kidney injury (AKI) induced by gentamicin (Morales et al., 2010) and CKD using 5/6 nephrectomy (Satriano et al., 2013), unilateral ureteral obstruction (UUO) (Cavaglieri et al., 2015), and adenine diet (Neven et al., 2018). Despite the potential benefits observed in animal studies, the clinical use of metformin is not recommended in patients with severe kidney dysfunction and absolutely contraindicated in those with an estimated glomerular filtration rate (eGFR) <30 ml/min/1.73 m^2^ because of the risk of lactic acidosis (Davies et al., 2018). Herein, we assessed the therapeutic effects of metformin on CKD MSCs and the applicability of autologous cell transplantation of metformin‐treated CKD MSCs to avoid the risk of adverse effects related to systemic metformin administration.

## 2 RESULTS

### 2.1 Metformin inhibits senescence of CKD MSCs

**FIGURE 1:**
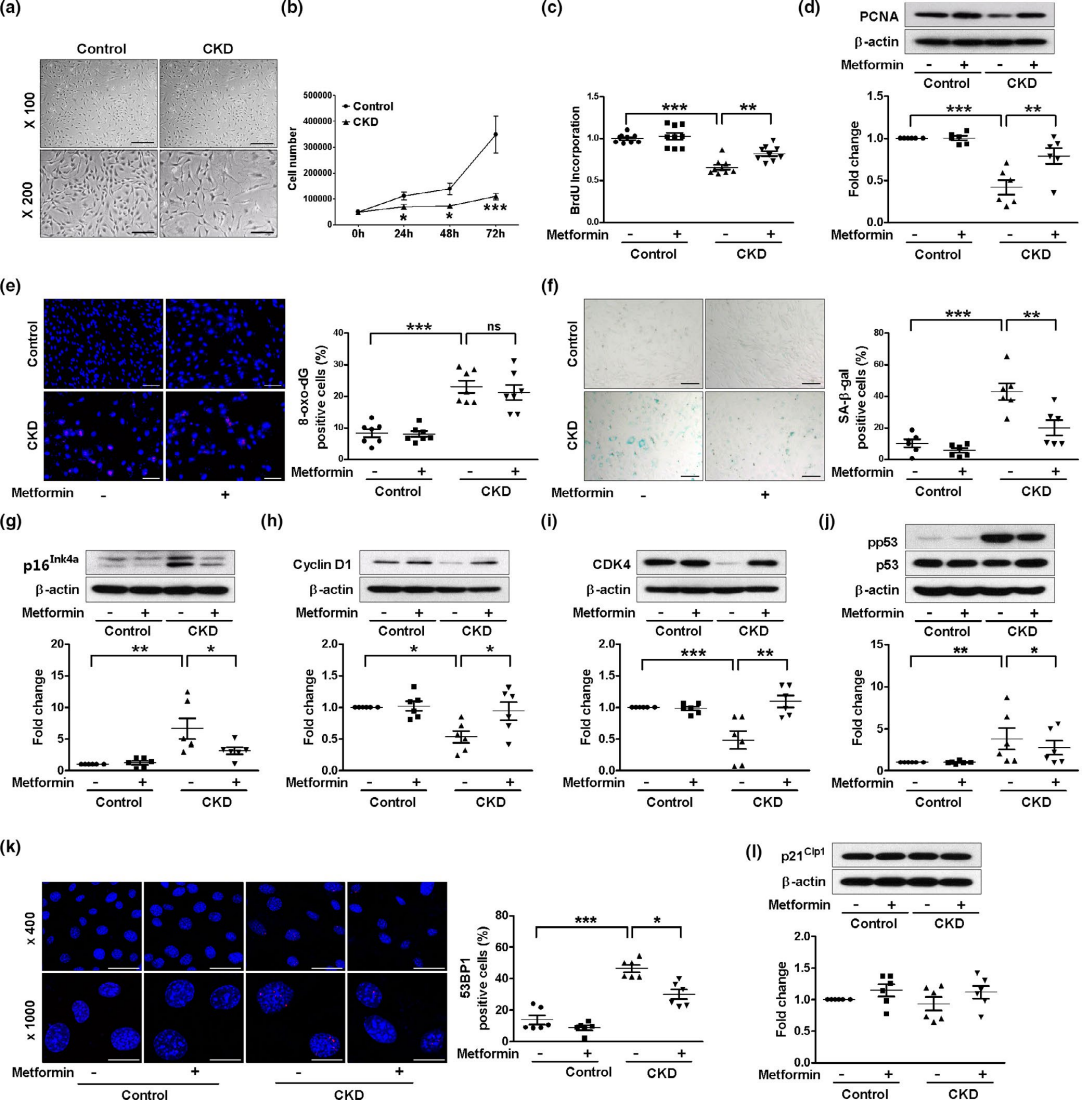
Metformin inhibits senescence of chronic kidney disease (CKD) mesenchymal stem cells (MSCs). (a) Representative bright field images of bone marrow‐derived MSCs isolated from sham‐operated or subtotal nephrectomized mice, scale bar, 200 μm and 100 μm. (b) Growth curve of control and CKD MSCs, *n* = 10. *p < 0.05 and ***p < 0.001 vs. corresponding control. (c–l) Control or CKD MSCs were treated with 10 μM metformin for 24 h. (c) Quantification of bromodeoxyuridine (BrdU) incorporation was shown, *n* = 9. Representative Western blots show protein levels of proliferating cell nuclear antigen (PCNA) (d), p16^Ink4a^ (g), cyclin D1 (h), cyclin‐dependent kinase 4 (CDK4) (i), phosphor p53 (j), and p21^Cip1^ (l), *n* = 6. Immunofluorescence staining of 8‐oxo‐2′‐deoxyguanosine (8‐oxo‐dG) (e, scale bar, 100 μm) and p53‐binding protein 1 (53BP1) (k, scale bar, 50 μm and 20 μm), *n* = 6–7. (f) Representative pictures of senescence‐associated‐β‐galactosidase (SA‐β‐gal)‐positive MSCs, scale bar, 100 μm, *n* = 6. *p < 0.05, **p < 0.01, and ***p < 0.001

We isolated MSCs from the bone marrow of sham‐operated or subtotal nephrectomized mice. These cells were previously characterized using surface markers (Noh et al., 2012), and their adipogenic or osteogenic differentiation capacity was confirmed as shown in Figure S1. CKD or metformin did not affect the differentiation capacity. CKD MSCs more frequently showed flat and enlarged morphologies as compared with spindle‐shaped control cells (Figure 1a), and their proliferation was significantly lower, as evident from growth curves, bromodeoxyuridine (BrdU) incorporation assay, and proliferating cell nuclear antigen (PCNA) expression (Figure 1b–d). As chronic inflammation and oxidative stress in CKD may lead to the accumulation of senescent cells, we measured the level of oxidation of guanosine residues by 8‐oxo‐2′‐deoxyguanosine (8‐oxo‐dG) immunofluorescence staining. As shown in Figure 1e, CKD MSCs had significantly higher expression level of 8‐oxo‐dG than control MSCs, suggestive of oxidative DNA damage. Other senescence markers such as higher expression of senescence‐associated β‐galactosidase (SA‐β‐gal) and p16^Ink4a^ and lower expression of cyclin D1 and cyclin‐dependent kinase 4 (CDK4) also confirmed cellular senescence in CKD MSCs (Figure 1f–i). Furthermore, the levels of phosphorylated p53, a key regulator of senescence, and p53‐binding protein 1 (53BP1) were higher in CKD MSCs than in control MSCs (Figure 1j,k). However, the expression of p21^Cip1^, another effector of early cellular senescence, was not different between CKD MSCs and control MSCs (Figure 1l). Treatment with metformin significantly attenuated the effects of CKD on cell proliferation and senescence without affecting the level of CKD‐induced oxidative DNA damage (Figure 1c‐k, Figure S2).

**FIGURE 2:**
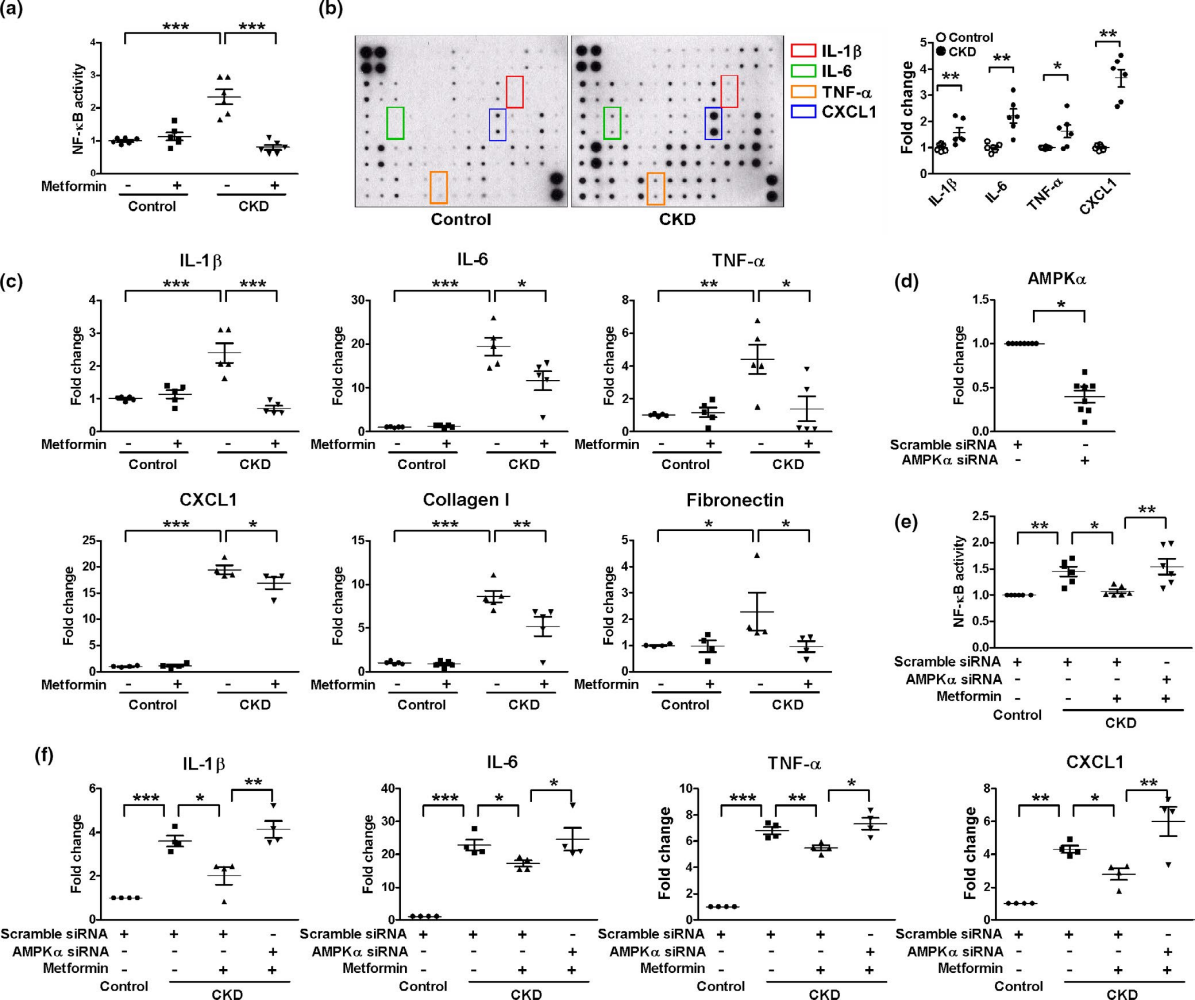
Metformin inhibits chronic kidney disease (CKD)‐induced NF‐κB activation and senescence‐associated secretory phenotype in adenosine monophosphate‐activated protein kinase (AMPK)‐dependent manner. Control or CKD MSCs isolated from sham‐operated or subtotal nephrectomized mice were treated with 10 μM metformin for 24 h. (a, e) NF‐κB activity was measured in nuclear protein extracts, *n* = 6. (b) Cytokine expression was evaluated in the supernatants of control or CKD MSCs, *n* = 3. (c, f) Real‐time RT‐PCR was performed to measure the mRNA levels of interleukin (IL)‐1β, IL‐6, tumor necrosis factor (TNF)‐α, chemokine (C‐X‐C motif) ligand 1 (CXCL1), collagen I, and fibronectin, *n* = 4–5. (d) Quantitative RT‐PCR analysis shows a decreased AMPKα mRNA level following siRNA transfection, *n* = 8. *p < 0.05, **p < 0.01, and ***p < 0.001

We next investigated nuclear factor‐kappa B (NF‐κB) activation and senescence‐associated secretory phenotype (SASP). CKD MSCs showed activation of NF‐κB and increased secretion of multiple proinflammatory cytokines (Figure 2a,b, Figure S3a). CKD‐induced NF‐κB activation, increased expression of proinflammatory SASP factors such as interleukin (IL)‐1β, IL‐6, tumor necrosis factor (TNF)‐α, and chemokine (C‐X‐C motif) ligand 1 (CXCL1), and a profibrotic phenotype with increased expression of collagen I and fibronectin were significantly attenuated by metformin treatment as shown in Figure 2a,c.

To investigate the involvement of the adenosine monophosphate‐activated protein kinase (AMPK) pathway in mediating the effect of metformin on NF‐κB activation and SASP, we determined the effect of metformin following small‐interfering RNA (siRNA)‐mediated AMPKα subunit expression knockdown, which abolished the effects of metformin (Figure 2d–f). Of note, AMPK pathway appears to be involved in suppression of NF‐κB activity and SASP even in basal state since siRNA‐mediated AMPKα knockdown enhanced those factors in the absence of metformin (Figure S3b,c).

### 2.2 Metformin improves the paracrine effects of CKD MSCs against lipopolysaccharide (LPS) or transforming growth factor‐beta 1 (TGF‐β1)‐induced renal tubular injury

**FIGURE 3:**
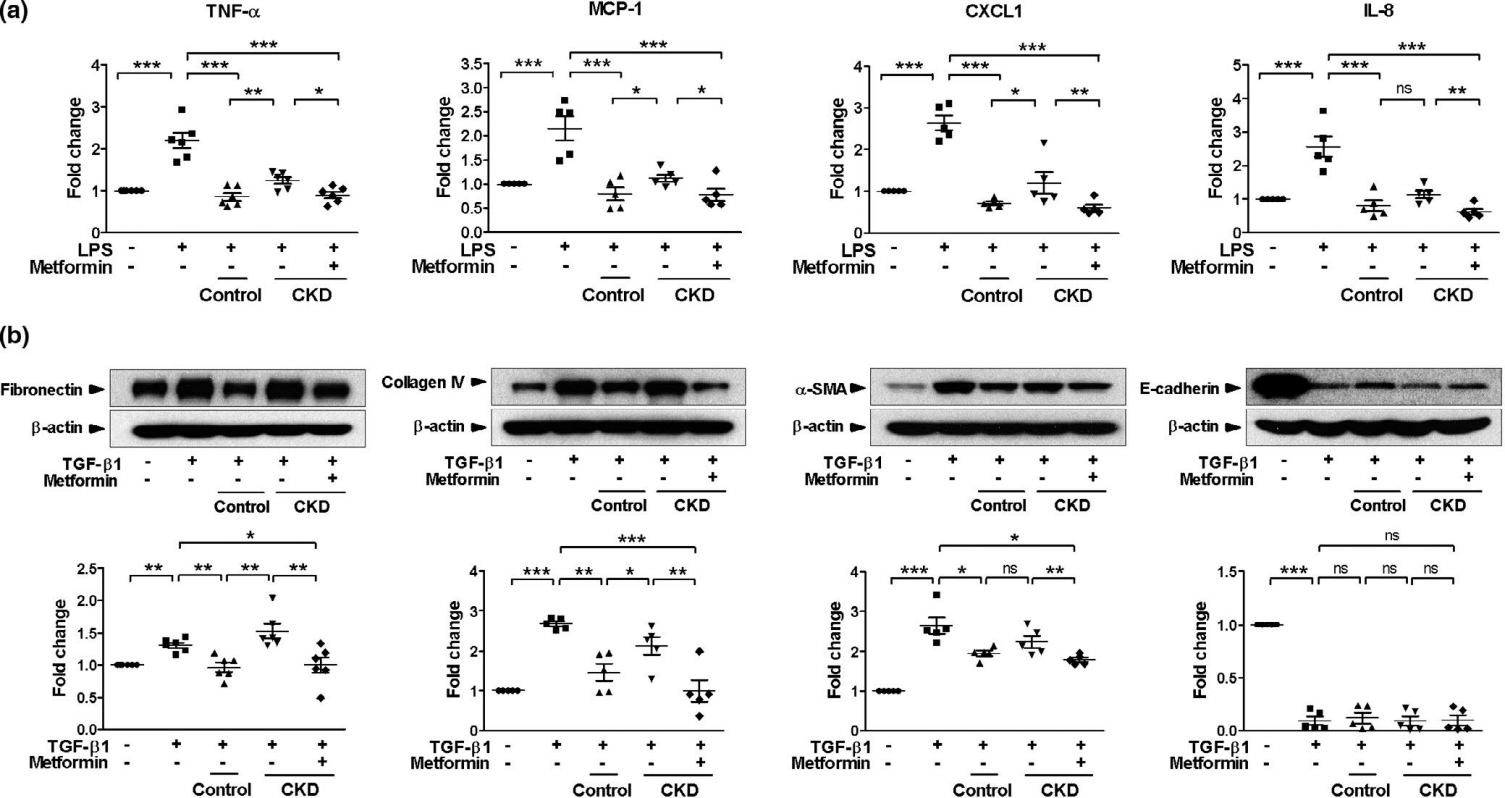
Metformin improves the paracrine effects of chronic kidney disease (CKD) MSCs against lipopolysaccharide (LPS) or transforming growth factor‐beta 1 (TGF)‐β1‐induced renal tubular injury. HK2 cells were co‐cultured with control or CKD MSCs with or without metformin (10 μM) and then stimulated with LPS (50 ng/ml) or TGF‐β1 (2 ng/ml) for 48 h. (a) Real‐time RT‐PCR was performed to measure the mRNA expression levels of tumor necrosis factor (TNF)‐α, monocyte chemoattractant protein (MCP)‐1, chemokine (C‐X‐C motif) ligand 1 (CXCL1), and interleukin (IL)‐8, *n* = 5–6. (b) Representative Western blots show protein levels of fibronectin, collagen IV, α‐smooth muscle actin (SMA), and E‐cadherin, *n* = 5–6. *p < 0.05, **p < 0.01, and ***p < 0.001

As MSCs are thought to mediate their regenerative effects via a paracrine mechanism, we investigated the influence of metformin on the impaired protective effects of CKD MSCs using a transwell system in the setting of LPS or TGF‐β1‐induced renal tubular injury model. HK2 cells were stimulated with LPS or TGF‐β1 and co‐cultured with control MSCs, CKD MSCs, or metformin‐treated CKD MSCs seeded on the insert of a transwell. HK2 cells exposed to LPS showed a marked increase in the expression of TNF‐α, monocyte chemoattractant protein‐1 (MCP‐1), CXCL1, and IL‐8 (Figure 3a). Co‐culture with control MSCs completely prevented the induction of cytokine and chemokine release from HK2 cells, whereas co‐culture with CKD MSCs was less effective. Metformin treatment significantly improved the anti‐inflammatory effect of CKD MSCs (Figure 3a). To further examine the effect of metformin on kidney fibrosis, we treated HK2 cells with TGF‐β1 and found that the expression of the proteins associated with kidney fibrosis, such as fibronectin, collagen IV, and α‐smooth muscle actin (SMA), was significantly elevated and that of E‐cadherin, a marker of epithelial cells, was downregulated. Co‐culture with control MSCs attenuated the increases in fibronectin, collagen IV, and α‐SMA. In comparison with CKD MSCs that showed less significant effects, metformin‐treated CKD MSCs showed better antifibrotic effects. However, co‐culture with either control or CKD MSCs failed to reestablish the expression of E‐cadherin in cells treated with TGF‐β1 (Figure 3b).

### 2.3 Metformin treatment improves the renoprotective effects of CKD MSCs in UUO kidneys

**FIGURE 4:**
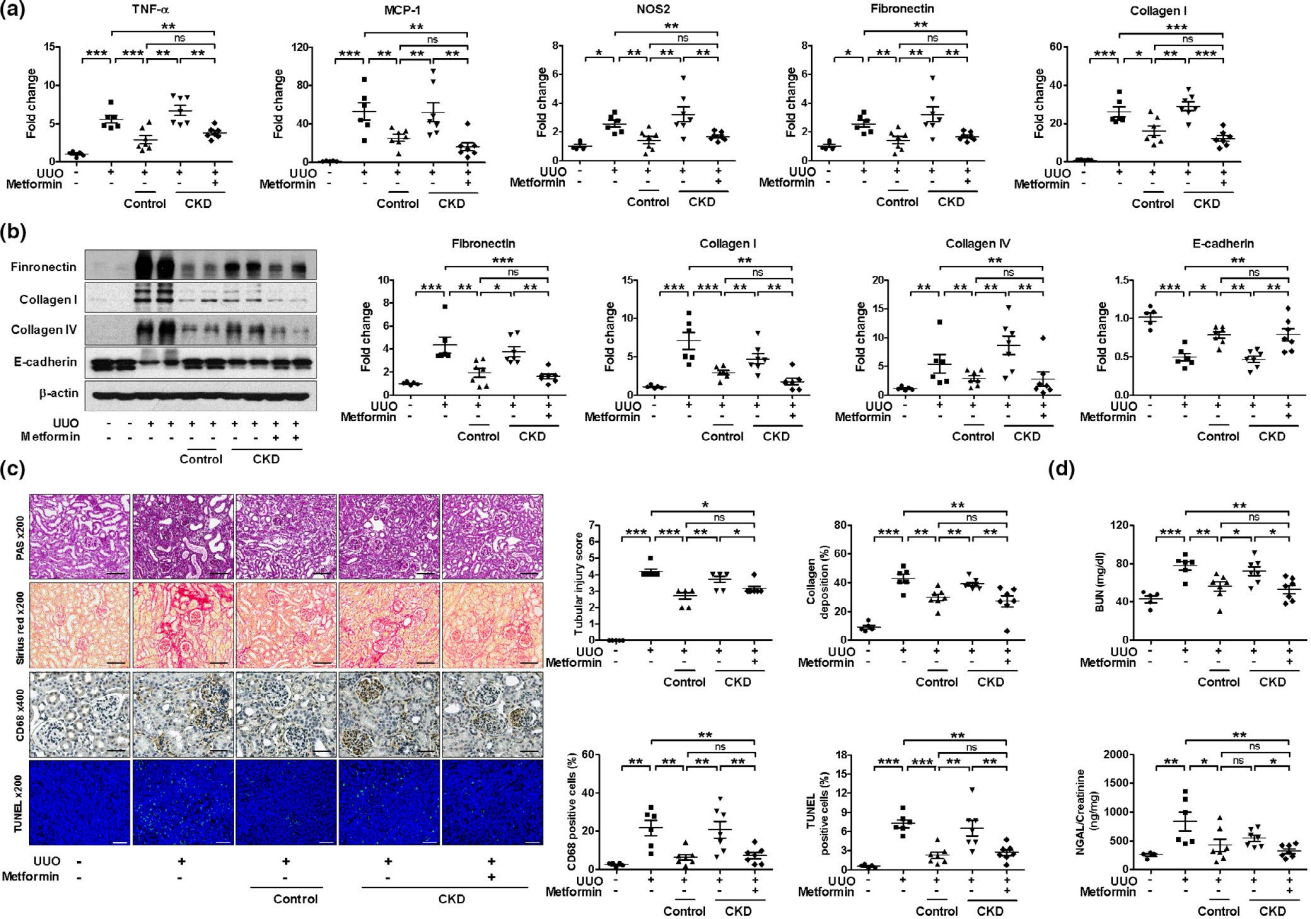
Metformin treatment improves the renoprotective effects of chronic kidney disease (CKD) MSCs in unilateral ureteral obstruction (UUO) kidneys. At the time of UUO surgery, 2 × 10^6^ control or CKD MSCs with or without metformin preconditioning for 24 h were intraperitoneally injected into each mouse. (a) Real‐time RT‐PCR was performed to measure the mRNA expression of tumor necrosis factor (TNF)‐, monocyte chemoattractant protein (MCP)‐1, nitric oxide synthase (NOS) 2, fibronectin, and collagen I. (b) Fibronectin, collagen I and IV, and E‐cadherin protein expression levels were analyzed using Western blot analyses. (c) Representative kidney sections stained with periodic acid–Schiff (scale bar, 100 μm), Picro Sirius Red (scale bar, 100 μm), immunohistochemical staining for CD68‐positive macrophages (scale bar, 50 μm), and terminal deoxynucleotidyl transferase dUTP nick end labeling (TUNEL) staining (scale bar, 100 μm). *n* = 5–7 for each group. *p < 0.05, **p < 0.01, and ***p < 0.001

To understand the therapeutic potential of metformin‐treated CKD MSCs in alleviating structural and functional damage as compared to control or CKD MSCs, we used a mouse model of kidney fibrosis induced by UUO. Transplanted MSCs were occasionally found in injured mouse renal tissues (Figure S4). The kidneys of C57Bl/6J mice subjected to UUO showed higher mRNA expression of proinflammatory cytokines such as TNF‐α, MCP‐1, and nitric oxide synthase 2 (NOS2) and profibrotic genes such as those encoding fibronectin and collagen I. The transcript levels of these genes were significantly decreased in groups injected with control MSCs or metformin‐treated CKD MSCs (Figure 4a). The changes in fibronectin and collagen I/IV expression and the effect of control and metformin‐treated CKD MSCs as compared to that of CKD MSCs were further confirmed by Western blotting. E‐cadherin expression showed an opposite trend (Figure 4b). Histological analysis indicated that the transplantation of control MSCs significantly attenuated tubular atrophy, tubular dilation, interstitial fibrosis, and infiltration of CD68‐positive cells and terminal deoxynucleotidyl transferase dUTP nick end labeling (TUNEL)‐positive apoptotic cells as compared to that of vehicle‐treated UUO kidneys (Figure 4c). Transplantation of CKD MSCs, however, showed less significant effects, which were rescued by metformin pretreatment. Deterioration of renal function, as evident from elevated blood urea nitrogen (BUN) and urinary neutrophil gelatinase‐associated lipocalin (NGAL) to creatinine ratio, was alleviated following transplantation of control MSCs but not CKD MSCs; metformin pretreatment showed a tendency toward better preservation of renal function as compared with CKD MSC treatment (Figure 4d).

### 2.4 Metformin inhibits CKD‐induced senescence and DNA damage in human retroperitoneal adipose tissue‐derived MSCs (ADMSCs)

**FIGURE 5:**
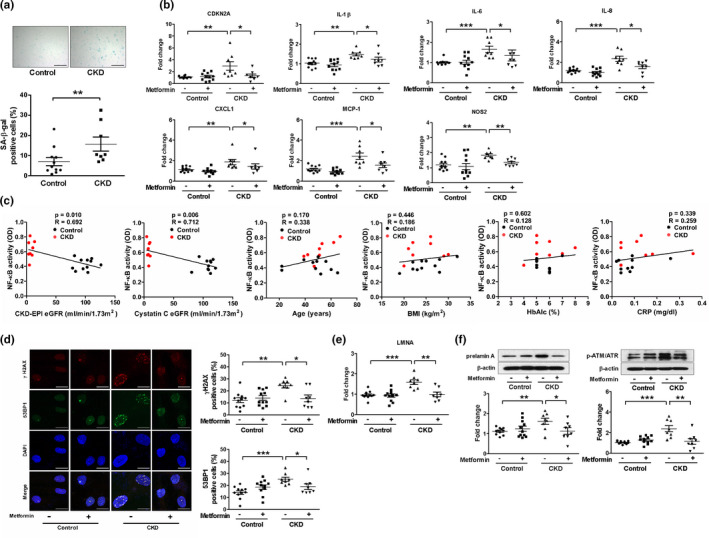
Metformin inhibits cellular senescence and DNA damage in retroperitoneal adipose tissue‐derived MSCs (ADMSCs) from patients with chronic kidney disease (CKD). Retroperitoneal adipose tissues were obtained from healthy kidney donors or patients with CKD during kidney transplantation. (a) Representative pictures of senescence‐associated‐β‐galactosidase (SA‐β‐gal)‐positive MSCs, scale bar, 200 μm. (b, e) Real‐time RT‐PCR was performed to measure the mRNA expression of cyclin‐dependent kinase inhibitor 2A (CDKN2A), interleukin (IL)‐1β, IL‐6, IL‐8, chemokine (C‐X‐C motif) ligand 1 (CXCL1), monocyte chemoattractant protein (MCP)‐1, nitric oxide synthase (NOS) 2, and LMNA. (c) NF‐κB activity was measured in nuclear protein extracts. NF‐κB activity of ADMSCs correlates with creatinine or cystatin C‐based estimated glomerular filtration rate (eGFR) but does not with age, body mass index (BMI), hemoglobin A1c (HbA1c), or C‐reactive protein (CRP). (d) Representative immunofluorescence staining for phosphorylation of the histone H2A variant H2AX (γH2AX) and p53‐binding protein 1 (53BP1). Scale bar, 20 μm. (f) Representative Western blots show protein levels of prelamin A and phosphor ataxia telangiectasia mutated (ATM)/ATM‐ and Rad3‐related (ATR). *n* = 11 biologically independent samples for control ADMSCs and *n* = 8 for CKD ADMSCs. *p < 0.05, **p < 0.01, and ***p < 0.001

The clinical significance of CKD‐associated senescence in MSCs was examined using ADMSCs from healthy kidney donors and patients with CKD. The clinical demographics of the subjects are shown in Table 1. The expression of SA‐β‐gal and cyclin‐dependent kinase inhibitor 2A (*CDKN2A*) gene encoding p16^Ink4a^ was higher in the ADMSCs from patients with CKD than in those from control subjects, confirming the CKD‐associated cellular senescence in human MSCs (Figure 5a,b). Furthermore, we found a significant correlation between NF‐κB activity and creatinine or cystatin C‐based eGFR. Thus, reduced renal function correlated with the activation of NF‐κB in ADMSCs, as observed in the MSCs from the bone marrow of rodents. In contrast, NF‐κB activity was not correlated with age, body mass index, hemoglobin A1c, or C‐reactive protein (Figure 5c). SASP, including production of IL‐1β, IL‐6, IL‐8, CXCL1, MCP‐1, and NOS2, significantly increased in ADMSCs from patients with CKD (Figure 5b). As the level of the DNA damage marker nuclear 53BP1 was significantly higher in the ADMSCs from patients with CKD (Figure 5d) and in the bone marrow MSCs from CKD mice (Figure 1k), we evaluated the upstream DNA damage response (DDR). Interestingly, the expression of *LMNA* gene and prelamin A, which has been proposed to interfere with DNA damage repair signaling to initiate early senescence in vascular cells, increased in CKD ADMSCs (Figure 5e,f). We also observed an increase in the phosphorylation of the histone H2A variant H2AX (γH2AX), a marker of DNA damage, and ataxia telangiectasia mutated (ATM)/ATM‐ and Rad3‐related (ATR), key regulators of DDR (Figure 5d,f). Metformin treatment for 72 h attenuated the expression of *LMNA* gene and prelamin A and DDR signaling as well as the senescent features of CKD ADMSCs, including upregulated *CDKN2A* expression and SASP (Figure 5b,d–f).

**TABLE 1:** Characteristics of the participants.

Variables	Healthy donors (*n* = 11)	CKD patients (*n* = 8)	*p* value
Age (years)	46.3 ± 13.9	51.5 ± 9.4	0.370
Gender (male *n*, %)	3 (27.3%)	6 (75.0%)	0.070
Hypertension (yes *n*, %)	2 (18.2%)	8 (100.0%)	0.001
Diabetes mellitus (yes *n*, %)	1 (9.1%)	4 (50.0%)	0.111
Body mass index (kg/m^2^)	24.9 ± 3.4	24.7 ± 3.8	0.913
Serum creatinine (mg/dl)	0.78 ± 0.14	8.44 ± 3.45	<0.001
CKD‐EPI eGFR (ml/min/1.73 m^2^)	98.77 ± 14.43	7.41 ± 3.58	<0.001
Cystatin C (mg/L)	0.80 ± 0.09	6.07 ± 1.22	<0.001
Cystatin C eGFR (ml/min/1.73 m^2^)	108.94 (106.26–113.00)	7.64 (6.26–9.63)	<0.001
HbA1c (%)	5.55 ± 0.51	5.80 ± 1.33	0.655
CRP (mg/dl)	0.07 (0.04–0.09)	0.12 (0.04–0.17)	0.152

Note

Mean ± standard deviation or median (interquartile range).

Abbreviations: CKD, chronic kidney disease; CKD‐EPI, CKD Epidemiology Collaboration; CRP, C‐reactive protein; eGFR, estimated glomerular filtration rate; HbA1c, hemoglobin A1c.

## 3 DISCUSSION

In the present study, we demonstrate the increased senescence of CKD MSCs that may contribute to poor regenerative potential and suggest metformin preconditioning as an effective strategy to overcome the senescence‐associated barrier to autologous patient‐derived MSC therapy in CKD. The actions of metformin against senescence were confirmed by multiple observations, including inhibition of SA‐β‐gal activity, p16^Ink4a^ expression, and activation of p53 and NF‐κB, resulting in a decreased expression of several genes related to SASP. Furthermore, we show that metformin decreased CKD‐induced prelamin A accumulation and DNA damage signaling, which may lead to cellular senescence. The renoprotective properties of CKD MSCs were significantly enhanced following metformin pretreatment in co‐culture experiments and in UUO model.

MSC senescence is associated with impairment in their regenerative potential. Senescent MSCs show limited proliferation, decreased differentiation potential, and impaired migratory and homing ability, and affect neighboring cell and tissue functions by producing factors related to SASP (Gnani et al., 2019; Hickson et al., 2016). As accumulation of oxidative stress is the key mechanism underlying cellular senescence (He & Sharpless, 2017; McHugh & Gil, 2017), it is likely that CKD MSCs exhibit increased senescence. Our data using mouse bone marrow MSCs revealed enhanced oxidative DNA damage and cellular senescence associated with CKD. Furthermore, as in rodents, accelerated senescence was confirmed in ADMSCs isolated from patients with CKD as compared to those from healthy kidney donors by the findings of higher expression of DNA damage markers γH2AX and 53BP1 as well as *CDKN2A* and higher percentage of SA‐β‐gal‐positive cells resulting in the upregulation in SASP genes. Our observations showing that CKD causes MSCs to undergo cellular senescence are in accordance with the findings that uremic toxin or hyperphosphatemia can induce senescence of vascular smooth muscle cells (Muteliefu et al., 2012; Takemura et al., 2011; Troyano et al., 2015) or myoblasts (Sosa et al., 2018), which have been suggested as potential mechanisms of cardio‐renal cross talk (Hénaut et al., 2018; Kaesler et al., 2020) or sarcopenia associated with CKD (Sosa et al., 2018).

Growing evidence has implicated nuclear lamina dysfunction in vascular senescence (Liu et al., 2013; Mattout et al., 2006; Ragnauth et al., 2010). The nuclear lamina is a protein meshwork that underlies the nuclear envelope, plays an important role in maintaining nuclear integrity, and is involved in diverse cellular functions. Lamins are the major components of the nuclear lamina, and mutations in *LMNA* gene encoding lamins have been implicated in various premature aging disorders such as Hutchinson–Gilford progeria syndrome (HGPS) (Eriksson et al., 2003). Prelamin A, a precursor protein of lamin A, undergoes stepwise post‐translational modifications, including farnesylation and proteolytic cleavage mediated by zinc metalloproteinase STE24 homologue, ZMPSTE24, to produce lamin A (Sinensky et al., 1994). Previous studies have shown that the accumulation of prelamin A in the vasculature results in the induction of vascular calcification and senescence (Liu et al., 2013) and that cardiac‐specific prelamin A accumulation induces inflammatory cardiomyopathy with premature senescence (Brayson et al., 2019). In the present study, we revealed the higher expression of prelamin A in CKD ADMSCs and its association with DNA damage and cellular senescence for the first time. ATM and ATR are key DDR signaling components in mammalian cells. Although DDR generally protects against disease, its chronic hyperactivation can contribute to pathologic processes such as atherosclerotic cardiovascular disease or stem cell dysfunction (Jackson & Bartek, 2009). The findings of our study demonstrating the activation of the ATM/ATR pathway in CKD ADMSCs suggest enhanced DNA damage and DDR signaling under CKD conditions. It is unexpected that p21 protein expression was not upregulated in bone marrow MSCs from CKD mice, while DDR was activated as evident from elevated levels of phosphorylated p53 and 53BP1. A plausible explanation of this phenomenon is that posttranscriptional or post‐translational regulation of p21 (Jung et al., 2010) might be involved in our experimental conditions, which needs to be further investigated.

Upregulation in NF‐κB signaling is a master regulator of SASP in senescent cells (Chien et al., 2011), and NF‐κB is a major mediator of the anti‐aging or anti‐senescence effects of metformin. Using bioinformatic analysis of oncogene‐expressing senescence cells, Moiseeva et al. reported that NF‐κB pathway is the most relevant transcription factor altered during senescence and inhibited by metformin treatment (Moiseeva et al., 2013). In agreement, Sultuybek et al. revealed NF‐κB signaling as the target of the anti‐aging effect of metformin in dermal fibroblasts (Kanigur Sultuybek et al., 2019). In line with these observations, our data indicate that CKD MSCs had higher NF‐κB activity, and this effect was rescued by metformin treatment, resulting in a decrease in SASP.

Metformin is the most widely used anti‐diabetic drug and currently recommended as the first‐choice treatment in patients with type 2 diabetes. Aside from anti‐hyperglycemic effects, the role of metformin in metabolic and cellular processes such as inflammation, autophagy, oxidative damage, apoptosis, and senescence is now well documented (Foretz et al., 2014; Kulkarni et al., 2020). Although several studies have highlighted the renal protective effects of metformin (Cavaglieri et al., 2015; Kwon et al., 2020; Morales et al., 2010; Neven et al., 2018; Satriano et al., 2013), there is a concern that metformin use is associated with the risk of lactic acidosis in patients with CKD. Therefore, current clinical guidelines (Davies et al., 2018) suggest that metformin therapy should not be initiated in patients with eGFR <45 ml/min/1.73 m^2^ and should not be used in patients with eGFR <30 ml/min/1.73 m^2^. Our approach of preconditioning CKD MSCs with metformin before cell transplantation would be a clinically applicable method to enhance the regenerative potential of patient‐derived dysfunctional MSCs without the concern for lactic acidosis. The metformin concentration used in this experiment was similar to that detected in the serum of patients with type 2 diabetes treated with metformin (Foretz et al., 2014; Frid et al., 2010). To the best of our knowledge, this is the first study to reveal the role of metformin preconditioning in the functional improvement of CKD MSCs. An important question of whether metformin suppresses the development of senescent cells or reverses senescence should be examined in another independent studies.

While the molecular mechanism of action of metformin remains poorly understood, its primary action is to activate AMPK (Foretz et al., 2014). Here we demonstrated the involvement of AMPK in mediating the effects of metformin, as AMPKα suppression using siRNA significantly blocked the effects of metformin on the activation of NF‐κB and upregulation of SASP genes. The mechanisms driving prelamin A accumulation in CKD ADMSCs, which is rescued by metformin treatment, remain undetermined. Deposition of prelamin A has been observed in prematurely aged vessels of children on dialysis (Shroff et al., 2010) and in vascular smooth muscle cells cultured in media supplemented with calcium and phosphate (Liu et al., 2013), suggesting prelamin A accumulation under CKD conditions. A recent study demonstrated that metformin reduced the expression of progerin, a truncated form of prelamin A, by inhibiting the serine–arginine‐rich splicing factor SRSF1 in MSCs derived from patients with HGPS (Egesipe et al., 2016). Although we did not evaluate the change in SRSF1 level induced by metformin, our observations that the expression of progerin was not detected in either control or CKD ADMSCs with or without metformin treatment (data not shown) suggest the involvement of a different mechanism. More studies are warranted to study the mechanism underlying metformin‐mediated attenuation of prelamin A accumulation in ADMSCs, including regulatory factors responsible for prelamin A accumulation, such as ZMPSTE24.

A limitation of our study is that we could only test ADMSCs from patients with CKD stage 5 since it was technically difficult to obtain adipose tissues from patients with early stages of CKD. Future experiments will need to investigate whether ADMSCs from patients with early stages of CKD would be more responsive to metformin. Another limitation is that possible differences in the properties of bone marrow MSCs and ADMSCs should be taken into consideration. Although a previous study reported that no significant differences were observed for growth kinetics, multi‐lineage differentiation capacity, and gene transduction efficiency as well as cellular senescence according to their sources (De Ugarte et al., 2003), the effect of CKD on these characteristics has not been directly compared.

In conclusion, our study suggests that metformin preconditioning may exhibit a therapeutic benefit to target accelerated senescence of CKD MSCs. This can be applied to achieve adequate cell functionality for developing patient‐derived autologous MSC‐based therapeutics in patients with CKD. Further studies to assess the efficacy and feasibility of this approach are important for future investigations.

## 4 EXPERIMENTAL PROCEDURES

### 4.1 Animals

All animal studies were conducted following approval from the Institutional Care and Use Committee of the Soon Chun Hyang University Hospital, and complied with the National Institutes of Health Guidelines for the Care and Use of Experimental Animals. Male C57Bl/6J mice were obtained from Central Lab. Animal Inc. (Seoul, Korea) and housed in a pathogen‐free facility set on a 12‐h light‐dark cycle. Mice had free access to water and regular laboratory chow (Cargill Agri Purina, Gunsan, Korea). For isolation of CKD MSCs, a CKD model was established as previously described (Noh et al., 2012). In brief, C57Bl/6J mice, 5 weeks old, were subjected to electrocoagulation of the left kidney surface, leaving a small portion of the hilum intact. After 2 weeks, the animals underwent surgical ablation of the contralateral right kidney. Control animals received a sham operation. Six weeks after surgery, mice were sacrificed by exsanguination under tiletamine (15 mg/kg, Virbac Laboratories, Carros, France), zolazepam (15 mg/kg, Virbac Laboratories), or xylazine (10 mg/kg, Bayer Korea, Seoul, Korea) anesthesia. In some experiments, the UUO model was established. In brief, a mid‐abdominal incision was made under anesthesia and the left ureter was isolated and ligated. Sham‐operated mice were used as controls. At the time of UUO surgery, 2 × 10^6^ control or CKD MSCs with or without metformin (Sigma‐Aldrich, St. Louis, MO) preconditioning for 24 h were intraperitoneally injected into each mouse. After 1 week, the animals were sacrificed. For in vivo trafficking of administered MSCs, the cells were incubated with DiI (Molecular Probe, Eugene, OR) at a concentration of 1 μg/ml in phosphate‐buffered saline (PBS) for 5 min at 37°C before administration.

### 4.2 Isolation of bone marrow MSCs

Primary MSCs were isolated from the pooled bone marrow from three mice and cultured as previously described. In brief, the bone marrow was collected by flushing femurs and tibias with Hank's balanced salt solution (HBSS) and 2% fetal bovine serum (FBS, Gibco, Grand Island, NY). The cells were washed with HBSS supplemented with 2% FBS and plated in a 75 cm^2^ flask with Dulbecco's modified Eagle's medium (DMEM; Gibco) and 10% FBS. After 3 days, nonadherent cells were removed during medium change, and adherent cells were further cultivated in DMEM with 10% FBS. By passage 6–7, a homogenous population of MSCs was obtained. These cells were previously demonstrated to be positive for stem cell antigen‐1, CD44, and platelet‐derived growth factor receptor‐α and negative for CD45 and CD11b (Noh et al., 2012). For adipogenic differentiation, the cells were incubated in α‐MEM containing 10% fetal calf serum, 10% horse serum, 100 U/ml penicillin, 100 μg/ml streptomycin, 12 mM l‐glutamine, 5 μg/ml insulin, 1 μM dexamethasone, and 0.5 μM 3‐isobutyl‐1‐methylxanthine (Sigma‐Aldrich) for 3 weeks. For osteogenic differentiation, the cells were grown in the osteogenic medium (Life Technologies, Grand Island, NY) for 1 week. Adipocytes were stained with Oil Red O (Sigma‐Aldrich), and osteocytes were stained with Alizarin Red S (Sigma‐Aldrich). Three biologically independent MSC lines per group were obtained, and the cells at equivalent passages (between passages 6 and 11) were used for experiments.

### 4.3 Isolation of ADMSCs from human subjects

The study protocol was reviewed and approved by the Institutional Review Board of Soon Chun Hyang University Seoul Hospital (2017‐10‐016), and the study was performed in accordance with the Declaration of Helsinki. Informed consent was obtained from all subjects. Retroperitoneal adipose tissues were obtained from healthy kidney donors or patients with CKD stage 5 during kidney transplantation. After washing with PBS, the adipose tissue was finely minced using scissors and digested in the presence of collagenase (1 mg/ml; Sigma‐Aldrich) for 1 h on a shaking incubator at 37°C. Cell suspension was centrifuged at 200 *g* for 10 min. The upper adipocyte fraction was discarded, while the stromal vascular fraction that settled down was resuspended in an RBC lysis buffer, diluted in 2% bovine serum albumin in PBS, and filtered through a 100‐μm cell strainer (Corning Inc., Corning, NY). The cells were plated and cultured in DMEM with 10% FBS. The positive expression of CD73 (eBioscience, San Diego, CA), CD90 (Invitrogen, Rockford, IL), and CD105 (Invitrogen) and the negative expression of CD14 (Invitrogen) and CD31 (Invitrogen) were confirmed by flow cytometry (Beckman Coulter, Fullerton, CA) as shown in Figure S5. Their adipogenic, osteogenic, or chondrogenic differentiation under specific medium conditions was confirmed as described earlier (Yoon et al., 2020). ADMSCs between passages 2 and 4 were used for experiments.

### 4.4 Growth curve

Mouse bone marrow MSCs were seeded into six‐well culture plates at 5 × 10^4^ cells/well in complete media (10% FBS in DMEM). After growth for 24, 48, and 72 h, the number of cells was counted.

### 4.5 BrdU incorporation assay

BrdU incorporation was measured using a cell proliferation enzyme‐linked immunosorbent assay (ELISA) for BrdU (Roche, Mannheim, Germany) according to the manufacturer's instructions.

### 4.6 SA‐β‐gal assay

SA‐β‐gal assay was performed using a senescence detection kit (BioVision, Milpitas, CA) according to the manufacturer's instructions. In brief, cultured cells were washed with PBS and fixed at room temperature for 15 min in a fixation solution. Fixed cells were incubated for overnight with a SA‐β‐gal staining solution at 37°C. The percentage of cells positively stained for SA‐β‐gal was quantified.

### 4.7 Blood and urine chemistry

BUN and urine creatinine levels were measured using a colorimetric method on a Cobas 8000 analyzer (Roche). Urinary NGAL levels were measured using an ELISA kit (R&D Systems, Minneapolis, MN).

### 4.8 Co‐culture experiment

HK2 cells (American Type Culture Collection, Manassas, VA) were seeded at a density of 1 × 10^6^ cells in the lower chamber of a transwell system (Corning Inc., Kennebunk, ME). Control or CKD MSCs with or without metformin (10 μM) were seeded onto transwell inserts at a concentration of 2.5 × 10^5^ cells. One day later, the medium was replaced with serum‐free media supplemented with or without metformin. After 6 h, HK2 cells were treated with LPS (50 ng/ml, Sigma‐Aldrich) or TGF‐β1 (2 ng/ml, R&D Systems) for 48 h.

### 4.9 NF‐κB activity

NF‐κB activity in nuclear protein extracts was measured using a TransAM NF‐κB p65 protein assay (Active Motif, Carlsbad, CA).

### 4.10 Lactate dehydrogenase assay, cytokine array, siRNA, real‐time polymerase chain reaction (RT‐PCR), Western blot analysis, and histology

Details were described in Appendix S1.

### 4.11 Statistical analyses

Data are presented as the mean ± standard error (SE) unless otherwise specified. Normality was assessed using GraphPad Prism 5 (GraphPad Software, San Diego, CA) Kolmogorov–Smirnov test. For experiments with more than 2 conditions, differences between groups were evaluated using one‐way analysis of variance followed by Bonferroni post hoc tests. When only 2 groups were compared, either an unpaired two‐tailed *t* test or Mann–Whitney test was used. A value of *p* < 0.05 was considered statistically significant.

## CONFLICT OF INTEREST

None.

## AUTHOR CONTRIBUTIONS

H Kim analyzed the data, provided critical discussion, and edited the manuscript. MR Yu performed the experiments. H Lee, SH Kwon, JS Jeon, and DC Han provided human samples and related data. H Noh designed the study, analyzed the data, and wrote and revised the manuscript.

## Supporting information

Supplementary Material

## Data Availability

The data that support the findings of this study are available from the corresponding author upon reasonable request.
