# Supplementary material for: Metformin inhibits chronic kidney disease‐induced DNA damage and senescence of mesenchymal stem cells
Source: Aging Cell. 2021 Feb 1;20(2):e13317. doi: 10.1111/acel.13317 (PMC7884040; doi:10.1111/acel.13317)
Supplement: Supplementary file 1 — Supplementary Material [file ACEL-20-e13317-s001.docx]

**Metformin inhibits chronic kidney disease-induced DNA damage and senescence of mesenchymal stem cells**

Hyoungnae Kim, Mi Ra Yu, Haekyung Lee, Soon Hyo Kwon, Jin Seok Jeon, Dong Cheol Han, and Hyunjin Noh

Supplementary methods

Supplementary figure 1-5

**Supplementary methods**

**Lactate dehydrogenase (LDH) assay**

LDH concentration was measured using the LDH cytotoxicity detection kit (Abcam, Cambridge, UK) according to the manufacturer’s instructions.

**Cytokine array**

Mouse bone marrow MSCs were seeded into six-well culture plates at 5 × 10^5^ cells/well. Sub-confluent cells were incubated with serum-free media for 30 h to arrest and synchronize cell cycle. Supernatants were collected and cytokines were detected using a mouse cytokine array kit (Abcam) according to the manufacturer’s protocol.

**siRNA**

An effective predesigned siRNA specific for AMPKα (Applied Biosystems, Carlsbad, CA) was selected in a preliminary study. The sequence was as follows: sense 5ʹ-GCUAUACCAAGUGGAUAGUTT-3ʹ and antisense 5ʹ-ACUAUCCACUUGGUAUAGCTTG-3ʹ. Cells were transfected with siRNA (50 nM/well) using Lipofectamine RNAiMAX (Life Technologies, Carlsbad, NY) reagent under serum- and antibiotic-free conditions for 24 h.

**Real-time polymerase chain reaction (RT-PCR)**

Total RNA was prepared using TRIzol reagent (Life Technologies) and subjected to cDNA synthesis using a StepOne Plus Real-Time PCR System (Applied Biosystems). For PCR reactions, TaqMan Assays-on-Demand Gene Expression Products from Applied Biosystems were used as follows: IL-1β, Mm00434228_m1 and Hs01555410_m1; IL-6, Mm00446190_m1 and Hs00985639_m1; TNF-α, Mm0043258_m1 and Hs00174128_m1; CXCL1, Mm04207460_m1 and Hs00605382_Gh; IL-8, Hs01567913_g1; MCP-1, Mm00441242_m1 and Hs00234140_m1; NOS2, Mm00440502_m1 and Hs01075529_m1; fibronectin Mm0156744_m1; collagen I, Mm00801666_g1; AMPKα, Mm01296700_m1; CDKN2A, Hs00923894_m1; and LMNA, Hs00153462_m1. As an internal control, 18S ribosomal RNA expression was quantified with the target genes using the gene expression products Mm03928990_g1 and Hs99999901_s1.

**Western blot analysis**

Tissue and cell lysates were subjected to Western blot analysis as per the standard procedure. Membranes were immunoblotted with antibodies against PCNA (Abcam, Cambridge, UK), p16^Ink4a^ (Abcam), cyclin D1 (Cell Signaling Technology, Danvers, MA), CDK4 (Cell Signaling Technology), phosphor or total p53 (Cell Signaling Technology), p21^Cip1^ (Cell Signaling Technology), collagen I/IV (Southern Biotech, Birmingham, AL), fibronectin (Abcam), E-cadherin (BD Bioscience, San Jose, CA), α-SMA (Sigma-Aldrich), prelamin A (Millipore, Temecula, CA), phosphor ATM-ATR (Cell Signaling Technology), progerin (Abcam), and β-actin (Cell Signaling), followed by probing with appropriate secondary antibodies.

**Histology**

Paraffin-embedded sections (3 µm) were subjected to periodic acid-Schiff (PAS) and Picro Sirius Red staining (Abcam). On PAS-stained kidney sections, more than 50 different cortical fields were randomly sampled to score tubular injury using Image Scope software (Aperio, Vista, CA). The tubules were evaluated according to the following scoring system: 0, no tubular injury; 1, ≤ 10% tubules injured; 2, 11%–25% tubules injured; 3, 26%–50% tubules injured; 4, 51%–74% tubules injured; and 5, ≥ 75% tubules injured (Takaori, K et al., 2016). Collagen deposition was determined on the Picro Sirius Red-stained kidney sections. The positive area was quantitatively measured using Image Scope software. For analysis of macrophage infiltration, paraffin-embedded sections were stained for CD68 (Abcam). Diaminobenzidine was used for visualization of immunoreactivity and hematoxylin was used for nuclear counterstaining.

Immunofluorescence staining was performed using paraffin-embedded sections (3 µm). After blocking with 10% normal goat serum for 1 h, the sections were incubated for overnight at 4°C with anti-8-oxo-dG (Trevigen, Gaithersburg, MD), H2AX (Abcam), and fluorescein isothiocyanate (FITC)-conjugated 53BP1 (Novus, Centennial, CO) antibodies. Antibody staining was visualized with Alexa Fluor 568 goat anti-rabbit or Alexa Fluor 488 goat anti-rabbit (Invitrogen), as appropriate. Cell nuclei were counterstained with 4′,6-diamidino-2-phenylindole (DAPI; Sigma-Aldrich) for 10 min. Slides were viewed using a confocal microscope (LSM 700, Carl Zeiss, Oberkochen, Germany). Apoptotic cells were detected in paraffin-embedded kidney sections using an In Situ Cell Death Detection Kit (Roche, Mannheim, Germany) according to the manufacturer’s instructions.

**References**

Takaori, K. et al. Severity and frequency of proximal tubule injury determines renal prognosis. *J Am Soc Nephrol* **27**, 2393-2406 (2016).


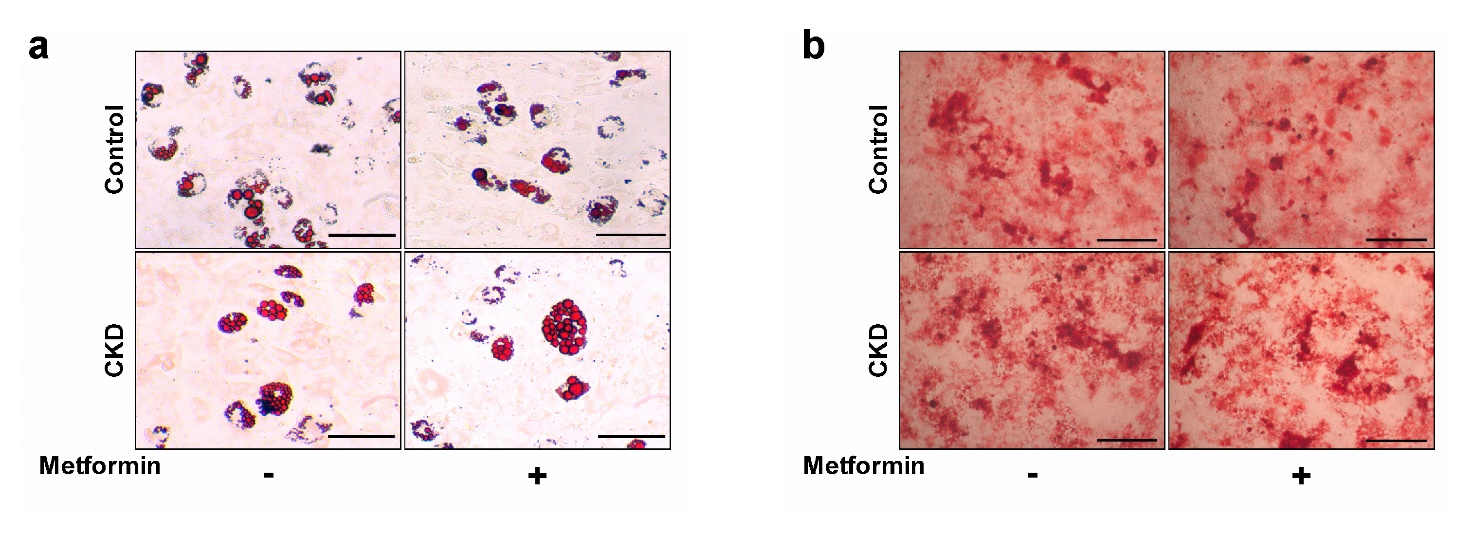


**Supplementary figure 1.** Differentiation capacities of control and chronic kidney disease (CKD) mesenchymal stem cells (MSCs) were confirmed by positive differentiation into adipocytes stained with oil red O (**a,** scale bar, 200 μm) and osteocytes stained with Alizarin Red S (**b,** scale bar, 500 μm).

**
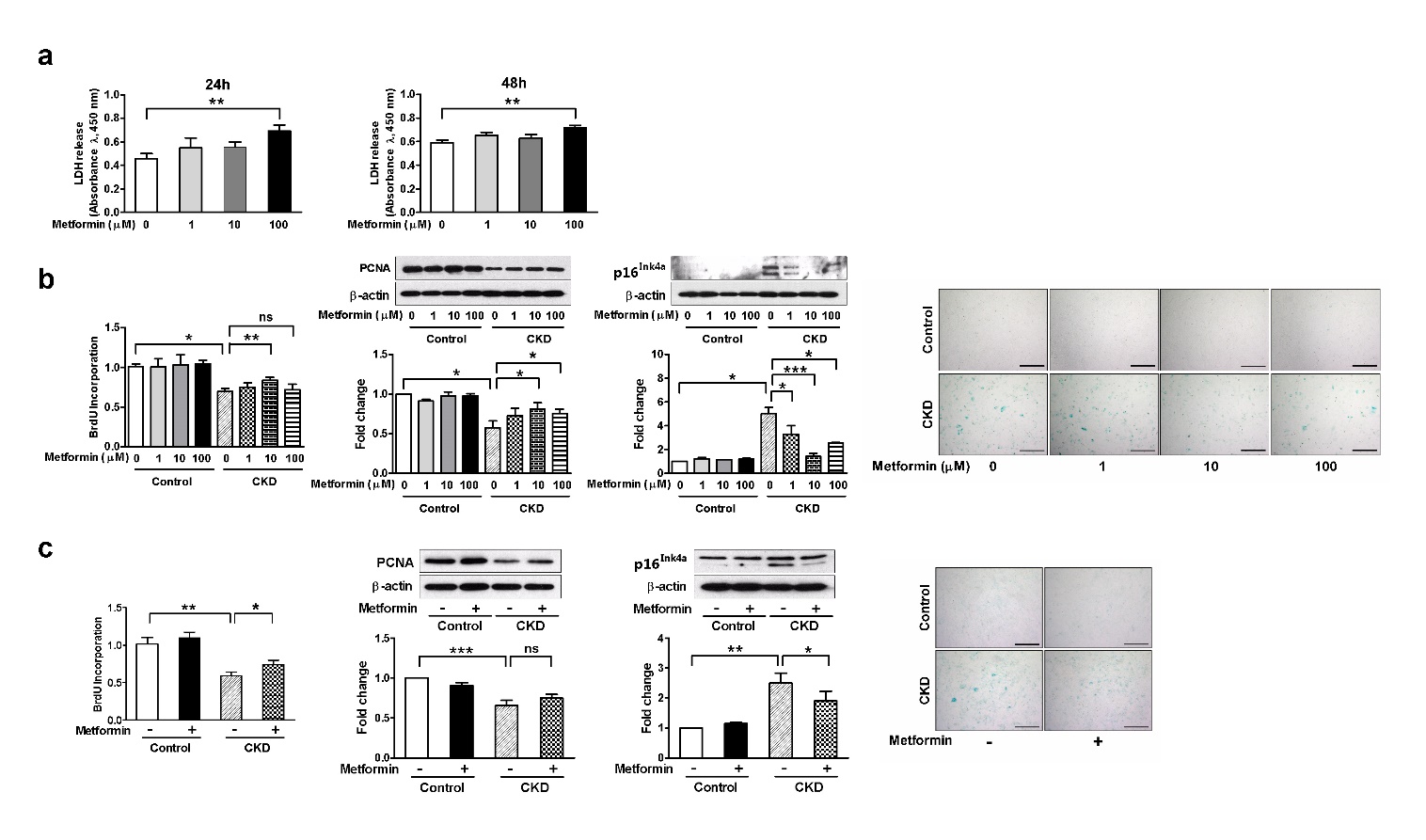
**

**Supplementary figure 2.** Metformin attenuates the effects of chronic kidney disease (CKD) on cell proliferation and senescence. (**a**) Toxicity of metformin assessed by lactic dehydrogenase (LDH) release. Control or CKD mesenchymal stem cells (MSCs) were treated with metformin at the indicated concentrations for 24 h (**b**) or at 10 μM concentration for 48 h (**c**). (**b,c**) Quantification of bromodeoxyuridine (BrdU) incorporation, representative Western blots showing protein levels of proliferating cell nuclear antigen (PCNA) and p16^Ink4a^, and representative pictures of senescence-associated-β-galactosidase positive MSCs were shown, scale bar, 200 μm, n=4-6. ^*^p < 0.05, ^**^p < 0.01, and ^***^p < 0.001.


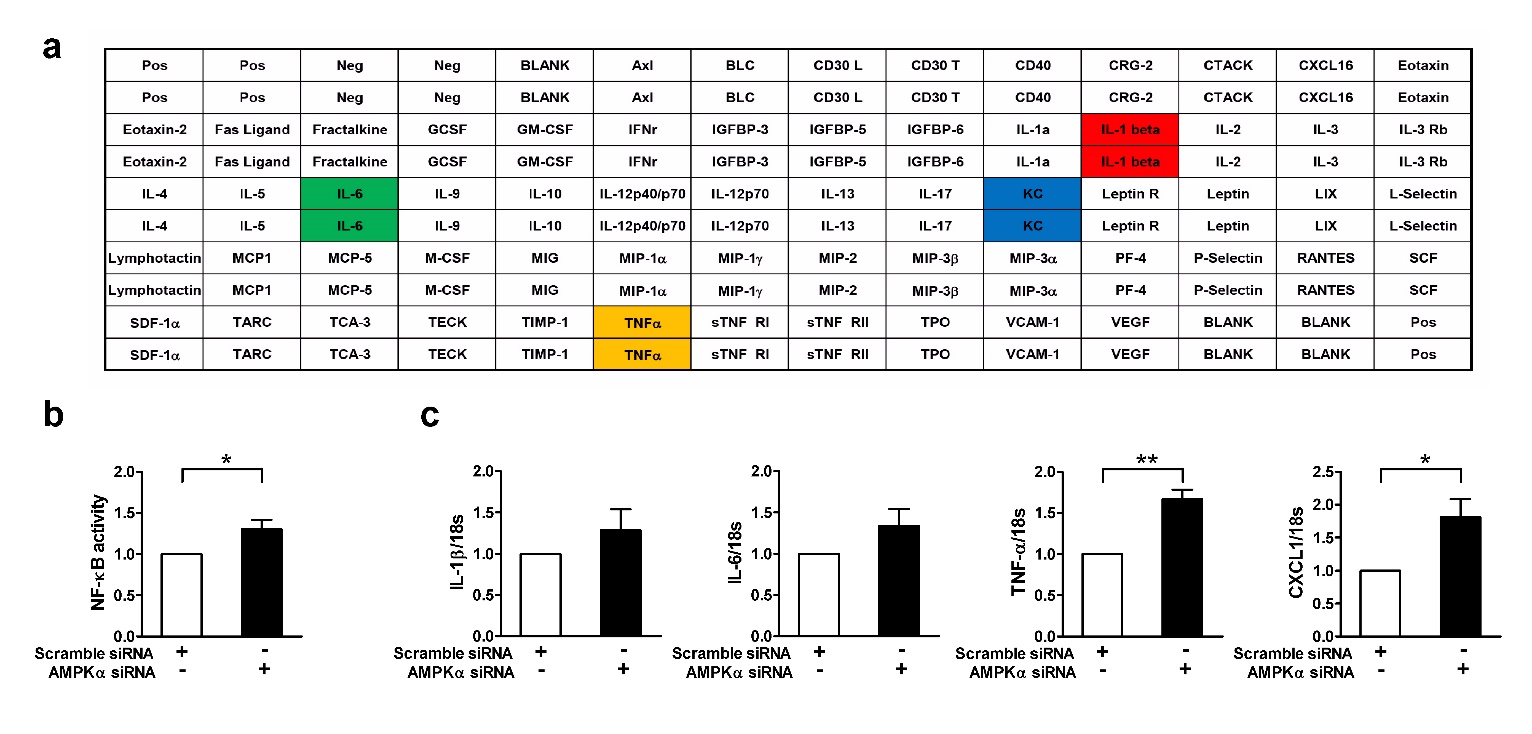


**Supplementary figure 3.** (**a**) Mouse cytokine array map. (**b,c**) Chronic kidney disease (CKD)-induced nuclear factor-kappa B (NF-κB) activation and increased expression of proinflammatory factors were further enhanced by small-interfering RNA (siRNA)-mediated adenosine monophosphate-activated protein kinase (AMPK) α knockdown in the absence of metformin. (**b**) NF-κB activity was measured in nuclear protein extracts of CKD mesenchymal stem cells (MSCs). (**c**) Real time RT-PCR was performed to measure the mRNA levels of interleukin (IL)-1β, IL-6, tumor necrosis factor (TNF)-α, and chemokine (C-X-C motif) ligand 1 (CXCL1) in CKD MSCs. n=5, ^*^p < 0.05 and ^**^p < 0.01.

**
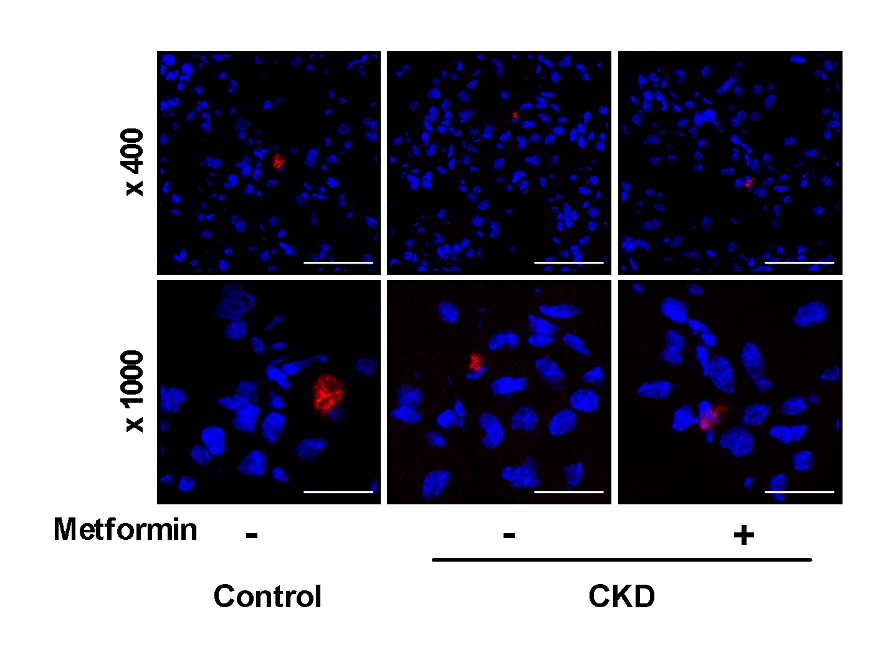
**

**Supplementary figure 4.** Representative image of renal tissue of unilateral ureteral obstruction (UUO) mouse at 7 days showing engrafted mesenchymal stem cells (MSCs) labeled with DiI (red). At the time of UUO surgery, 2 × 10^6^ control or chronic kidney disease (CKD) MSCs with or without metformin preconditioning for 24 h were intraperitoneally injected into each mouse. The presence of MSCs was determined with confocal microscope using 10 μm frozen sections. Nuclei were stained with DAPI. Scale bars, 50 μm and 20 μm.


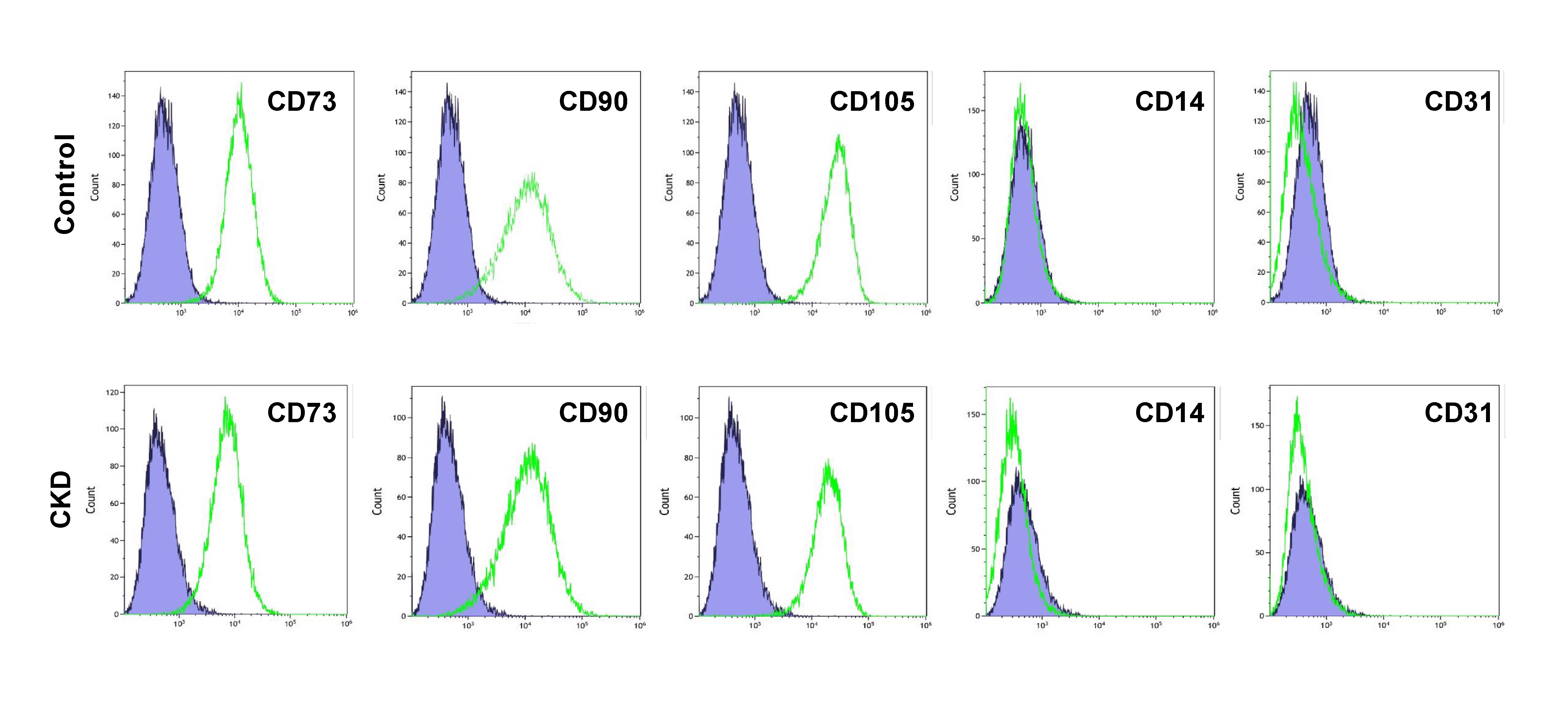

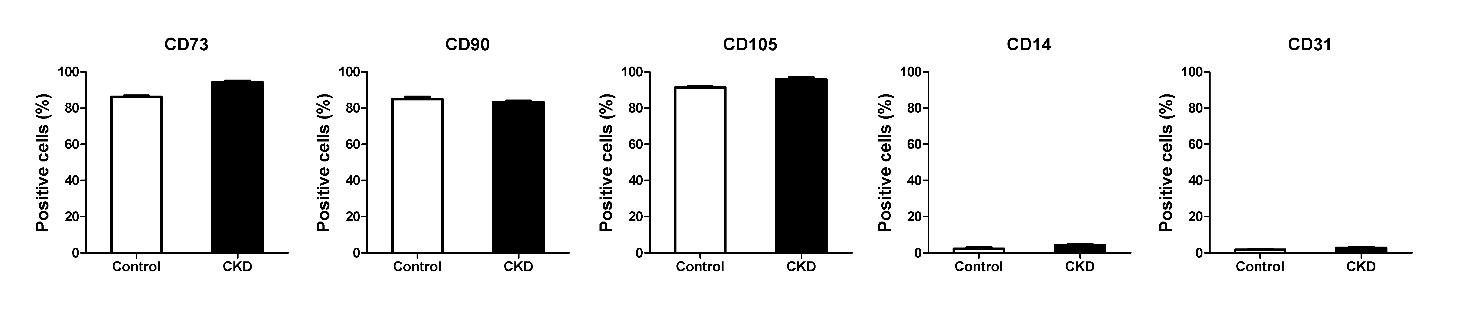


**Supplementary figure 5.** Representative flow cytometric plots of immunophenotypic characterization of retroperitoneal adipose tissue-derived mesenchymal stem cells from healthy kidney donors and patients with chronic kidney disease (CKD). Isotype-matched IgG was used as a control.
